# The Odontological Society of Pennsylvania

**Published:** 1889-11

**Authors:** 


### THE ODONTOLOGICAL SOCIETY OF PENNSYLVANIA

The attendance at the first two fall meetings has been large, and everything points to an active year's work. The committee appointed to consider the plan for the formation of a literary and scientific club have reported progress, but asked for another month to perfect their arrangements. The executive committee reported a plan for an additional course of lectures to be held under the auspices of the society, but to be given on evenings other than those of the regular society meeting. The first of these was given Saturday evening, November 2, by Professor Ernest Laplace, of Philadelphia, on the subject of "Fermentation: its Cause and Effects," and was listened to with much interest, as explaining the cause of decay and perhaps other oral diseases. Professor Laplace is widely known as the discoverer of the value of acid-sublimate in antiseptic surgery. He is thoroughly posted in microbiology, having spent several years abroad in the laboratories of Pasteur, the discoverer of fermentation, and Koch, of later but not less fame. The lecturer succeeded in simplifying the subject so as to bring it within the comprehension of all. The doctor has been invited to give another lecture, which will consist of a practical demonstration of the different ferments, with a description of the process of their cultivation, and the apparatus employed. For the latter the beautiful and complete apparatus which has just been ordered by the society from Germany for their new scientific club-rooms will be used.

Professor Harrison Allen, of Philadelphia, will deliver a lecture in the course on the catarrhal nature of oral affections, under the title of "Clinical Signs in Common to Nose, Throat, and Mouth." Professor Allen's extensive experience as a specialist in the treatment of affections of the nose, throat, and oral cavity, especially fits him for handling the subject assigned him.

Professor E. D. Cope, of Philadelphia, will give a lecture upon "The Evolution of the Mammalian Dentition." His wide reputation as a comparative anatomist and paleontologist is a sufficient guarantee that the subject will be treated in a masterly manner.

The course will be closed by Professor Sudduth, with a lecture on "The Morphology of the Micro-Organisms found in the Mouth," using the stereopticon to throw the photo-micrographs on the screen. The lectures will be free, and all are invited to attend.

We congratulate the readers of the International upon the action of the society, for the papers will belong to the Journal for publication.

